# Optimizing smoke alarm signals: Testing the effectiveness of children’s smoke alarms for sleeping adults

**DOI:** 10.1186/s40621-020-00279-6

**Published:** 2020-10-12

**Authors:** Gary A. Smith, Sandhya Kistamgari, Mark Splaingard

**Affiliations:** 1 Center for Injury Research and Policy, The Abigail Wexner Research Institute at Nationwide Children’s Hospital, 700 Children’s Drive, Columbus, OH 43205 USA; 2 Department of Pediatrics, The Ohio State University College of Medicine, Columbus, OH USA; 3 Child Injury Prevention Alliance, Columbus, OH USA; 4 Sleep Disorders Center, Nationwide Children’s Hospital, Columbus, OH USA

**Keywords:** Injury prevention, Fire and burns, Fire emergencies, Smoke alarm effectiveness, Sleep

## Abstract

**Background:**

Being asleep is an important risk factor for death during a residential fire; however, the high-frequency tone smoke alarms in many homes will not adequately awaken children who are old enough to self-rescue. In a series of previous studies, we identified smoke alarm signals that effectively awaken children 5–12 years old and prompt their escape. Because it is impractical to have separate alarms for children and adults in a household, the purpose of this study is to test whether alarms that are effective in awakening children and prompting their escape are also effective among adults.

**Methods:**

Using a randomized, non-blinded, repeated measures design, 150 adults 20–49 years old were exposed during stage 4 sleep to four different smoke alarms. Statistical tests included the Kaplan-Meier estimator, generalized Wilcoxon test, and hazard ratios with Wald’s 95% confidence intervals.

**Results:**

The median age of study subjects was 30.0 years and 67.3% were female. Almost all (*n* = 149) subjects awakened and performed the escape procedure to all four alarms; one individual did not awaken or escape to the high-frequency tone alarm. The median time-to-awaken was 2.0 s for the high-frequency tone alarm and 1.0 s for the other three alarms. The median time-to-escape for the high-frequency tone alarm was 12.0 s, compared with 10.0 s for the low-frequency tone alarm and 9.0 s each for the female and male voice alarms. All pairwise comparisons between the high-frequency tone alarm and each of the other three alarms were statistically significant for the probability functions for time-to-awaken and time-to-escape. There were no significant differences in these outcome measures between the latter three alarms, except for female voice versus low-frequency tone alarms for time-to-escape.

**Conclusions:**

All alarms performed well, demonstrating that smoke alarms developed for the unique developmental requirements of sleeping children are also effective among sleeping adults. Compared with a high-frequency tone alarm, use of these alarms may reduce residential fire-related injuries and deaths among children, while also successfully alerting adult members of the household.

## Background

Smoke alarms are a key component of the strategy to prevent residential fire-related injury and death. Arousing sleeping individuals and alerting them to the emergency in the event of a residential fire is important because the rate of fire-related mortality is three times higher during sleep, and about half of residential fire fatalities occur at night while the decedents are sleeping (Bruck and Ball 2007; Runyan et al. 1992). Although the high-frequency tone alarms found in many households awaken most adults, they are not effective in awakening children (Bruck and Horasan 1996; Busby et al. 1994; Nober et al. 1981; Smith et al. 2019; Smith et al. 2006; Underwriters Laboratories, Smoke alarm STP research group 2003). Our previous research tested key characteristics of smoke alarms with the goal of developing an alarm that can awaken children and prompt their escape (Smith et al. 2019; Smith et al. 2006; Smith et al. 2020a; 2020b). We initially demonstrated that an alarm using the voice of a child’s mother awakened 96% of children and prompted 83% to escape, significantly out-performing a high-frequency tone alarm (Smith et al. 2006). We subsequently showed that personalizing the voice alarm signal with the child’s first name or mother’s voice did not increase alarm effectiveness (Smith et al. 2019; Smith et al. 2020a). We then demonstrated that a low-frequency tone alarm and a female voice alarm each performed better than comparator alarm signals (Smith et al. 2020a). Additionally, we showed that alarms using a male voice, female voice, or a combination of a female voice and a low-frequency tone were each significantly more effective than a high-frequency tone alarm, but that there was no significant difference in effectiveness when compared with each other (Smith et al. 2020b). Now that effective smoke alarm signals for sleeping children have been identified, it is important to test their effectiveness among adults because it is impractical to have separate alarms for children and adults in a household.

This study tested smoke alarm signals among adults that have been previously shown to be effective among children 5–12 years of age. It tested whether adults will awaken from stage 4 slow wave sleep (S4S) and perform an escape procedure in response to a smoke alarm that uses a female voice, male voice, or low-frequency tone; a comparator high-frequency tone alarm was also included in the study. The findings of this study contribute to the identification of effective smoke alarms for children that are also effective for sleeping adults. This study promotes the goal of reducing residential fire-related injuries and deaths among children and adults.

## Methods

### Study population

The study population consisted of adults 20–49 years old, who were recruited via study announcements using institution-wide emails in a large academic children’s hospital and the hospital’s Facebook page. Individuals were eligible to enroll in the study if they 1) did not have a medical condition and weren’t taking a medication that might affect sleep, arousal, or their ability to perform the study’s escape procedure, 2) did not have a hearing impairment, 3) did not have an acute illness at the time of the study, and 4) spoke English. Participants received a pure-tone hearing screening test on the first night of the study using a Maico MA25 portable audiometer and had to successfully respond to all tested frequencies of 500, 1000, 2000, and 4000 Hz (Hz) at < 30 dB (dB) in both ears to be eligible to participate in the study.

This study did not include older adults. We are conducting a separate study in that population because of differences in the prevalence of slow wave sleep, hearing loss, and modifications of the escape procedure needed to ensure subject safety. In addition, previous research has shown that, compared with younger adults, individuals > 65 years old have lower or similar auditory arousal thresholds (AATs) for low-frequency tone and male voice alarms, respectively (Bruck et al. 2006). An AAT is the intensity level in dB of an auditory stimulus required to arouse an individual from sleep. Therefore, because young adults are more refractory to arousal than older adults to these alarms, if an alarm signal is successful in our current study, it would be expected to be at least as successful in a study among older adults.

The study sample size of 150 was based on the sample size used in our previous studies employing the same study design, which had demonstrated adequate statistical power (Smith et al. 2019; Smith et al. 2020a; 2020b). Among the 199 subjects initially enrolled, 49 withdrew because of the following reasons: 40 were unable to fall asleep, 5 did not attain S4S, 2 were bothered by the alarms, and 2 had a mild reaction to scalp preparation for electrodes. This yielded a final study sample of 150 individuals.

### Study design

This study used a randomized, non-blinded, repeated measures design to evaluate the ability of the study alarms to awaken individuals and prompt their performance of an escape procedure. Participants were each exposed during S4S of separate sleep cycles to these four smoke alarm signals: 1) female voice, 2) male voice, 3) low-frequency tone, and 4) high-frequency tone. The voice message used in the female and male voice alarms was “Fire! Fire! Wake up! Get out of bed! Leave the room!” The repeated measures design used by our study avoids potential confounding due to variation of AATs among individuals (inter-subject variability can be high) and takes advantage of the stability of AATs for an individual across sleep cycles (intra-subject variability is low) (Bonnet et al. 1978; Bruck 2001; Zepelin et al. 1984).

The female and male voice alarms and the low-frequency tone alarm used in this study have been shown to effectively awaken children 5–12 years old from S4S and prompt their performance of an escape procedure upon awakening (Smith et al. 2020a; 2020b). Although the low-frequency tone alarm was adopted as the United States standard for sleeping areas in 2014 (National Fire Protection Association 2016), a high-frequency (approximately 3200 Hz) tone alarm was also included in this study because it is the alarm type currently found in many homes. The low-frequency (500 Hz square wave) alarm employed in this study was a Simplex 1996, 4100 Fire Alarm and is the same alarm previously used in studies by Proulx and Laroche (2003), Bruck, et al. (1998), and our team (Smith et al. 2020a). Based on the Latin Square shown in Table 1, four sequences of alarm signals were used to minimize the possibility of a sequence effect. Block randomization (in blocks of four) of these sequences within each of three age groups (20–29, 30–39, 40–49 years old) was performed and then placed in sequentially numbered sealed envelopes by a research assistant, who was not involved with study enrollment or conducting the study. Study participants received the next available envelope for their age group upon arrival for their first study night, and only study staff knew the assigned alarm sequence after the envelope was opened. Alarm signals were amplified through small, smoke alarm-size speakers in the study bedrooms, which provided consistent signals at 85 dB when measured at the pillow. Study rooms were comfortably decorated to resemble a typical residential setting.

**Table 1:** Latin Square Showing the Four Alarm Signal Sequences

Alarm sequences	Night 1		Night 2	
	Sleep cycle 1	Sleep cycle 2	Sleep cycle 1	Sleep cycle 2
1	A	B	C	D
2	C	A	D	B
3	D	C	B	A
4	B	D	A	C

A, B, C, and D represent the four alarm signals used in the study

Subjects were taught an escape procedure on the night of the study prior to going to sleep; they were instructed to get out of bed when awakened by an alarm, walk to the bedroom door, and exit. Sleep stage was monitored to ensure that comparisons among alarm signals were not influenced by the sleep stage variability of AATs. After bedroom lights were turned off, continuous electroencephalography (EEG), electro-oculography, and chin electromyography via telemetry with synchronized low-light video monitoring were conducted by a polysomnography (PSG) technician. The EEG montage consisted of F3, F4, C3, C4, O1, O2, M1, and M2 electrodes.

### Testing protocol and measurements

Each study subject was allowed to progress into S4S and remained there for 5 min before an alarm was triggered. S4S is an older nomenclature for a deep stage of slow wave (N3) sleep and is defined as high voltage (> 75 microvolts peak-to-peak amplitude), slow wave (0.5–2 Hz) EEG activity accounting for more than 50% of a 30-s EEG/PSG epoch, measured over the frontal regions (Rechtschaffen and Kales 1968). Alarm signals were tested during S4S because it has the highest AAT, and therefore individuals in S4S are the most refractory to arousal (Underwriters Laboratories, Smoke alarm STP research group 2003). “Time-to-awaken” is the interval from the triggering of the alarm to the initiation of at least a 3-s arousal associated with movement and subsequent awake EEG. The interval from when the alarm was triggered until the study participant opened the bedroom door is the “time-to-escape.” If an alarm failed to awaken a subject after 5 min, the individual was awakened by research staff. This procedure was conducted during the first and second sleep cycles on two separate study nights at least 6 days apart, resulting in each subject being exposed to four different alarm signals (two different signals each night). Testing on consecutive nights was not done to avoid the possibility of confounding effects attributable to sleep deprivation and altered sleep architecture. A senior certified PSG technician determined the “time-to-awaken” from the EEG-video recordings, which was later reviewed and verified by one of the authors (M.S.), who is a physician board-certified in sleep medicine, while blinded to the alarm used. No discrepancies were identified during this review.

### Statistical analysis

Statistical analyses were performed using SAS 9.4 (SAS Institute Inc., Cary, NC). The Kaplan-Meier estimator was used to estimate the probability functions for time-to-awaken and time-to-escape, which were censored after 5 min. The generalized Wilcoxon test was used to assess the overall equality and pairwise comparisons of time-to-awaken and time-to-escape probability functions. Hazard ratios (HRs) with Wald’s 95% confidence intervals (CIs) were calculated for each pair of alarms. Statistical significance was determined at *p* < 0.05.

This study was approved by the institutional review board of the authors’ institution. Written informed consent was obtained from study participants. Participants were compensated monetarily for their time.

## Results

Among the 150 study subjects, the median age was 30.0 years (interquartile range [IQR]: 25.0–37.0) and 67.3% (*n* = 101) were female. All subjects awakened and performed the escape procedure to all four alarms, except one individual; a 47-year-old male did not awaken or escape to the high-frequency tone alarm but did so for the other three alarms (Table 2).

**Table 2:** Awakening and Escaping by Type of Alarm, Age Group, and Sex

	Number of Participants	Number Awakened	Time-to-Awaken (seconds)	Number Escaped	Time-to-Escape (seconds)
Type of Alarm, Age Group, Sex	n	n (%)	Median (IQR)	n (%)	Median (IQR)
**Female Voice**					
Age (years)					
20–29	69	69 (100.0)	1.0 (1.0 to 1.0)	69 (100.0)	9.0 (7.0 to 12.0)
30–39	53	53 (100.0)	1.0 (1.0 to 2.0)	53 (100.0)	9.0 (7.0 to 11.0)
40–49	28	28 (100.0)	1.0 (1.0 to 2.0)	28 (100.0)	9.0 (7.0 to 11.5)
Sex					
Male	49	49 (100.0)	1.0 (1.0 to 2.0)	49 (100.0)	9.0 (7.0 to 12.0)
Female	101	101 (100.0)	1.0 (1.0 to 1.0)	101 (100.0)	9.0 (7.0 to 12.0)
Subtotal	150	150 (100.0)	1.0 (1.0 to 2.0)	150 (100.0)	9.0 (7.0 to 12.0)
**Male Voice**					
Age (years)					
20–29	69	69 (100.0)	1.0 (1.0 to 1.0)	69 (100.0)	9.0 (8.0 to 13.0)
30–39	53	53 (100.0)	1.0 (1.0 to 2.0)	53 (100.0)	9.0 (8.0 to 10.0)
40–49	28	28 (100.0)	1.0 (1.0 to 1.5)	28 (100.0)	9.0 (7.0 to 12.0)
Sex					
Male	49	49 (100.0)	1.0 (1.0 to 2.0)	49 (100.0)	8.0 (7.0 to 11.0)
Female	101	101 (100.0)	1.0 (1.0 to 1.0)	101 (100.0)	9.0 (8.0 to 12.0)
Subtotal	150	150 (100.0)	1.0 (1.0 to 2.0)	150 (100.0)	9.0 (7.0 to 12.0)
**Low-Frequency Tone**					
Age (years)					
20–29	69	69 (100.0)	1.0 (1.0 to 1.0)	69 (100.0)	9.0 (7.0 to 14.0)
30–39	53	53 (100.0)	1.0 (1.0 to 1.0)	53 (100.0)	11.0 (8.0 to 13.0)
40–49	28	28 (100.0)	1.0 (1.0 to 1.0)	28 (100.0)	10.0 (7.0 to 11.5)
Sex					
Male	49	49 (100.0)	1.0 (1.0 to 2.0)	49 (100.0)	9.0 (7.0 to 13.0)
Female	101	101 (100.0)	1.0 (1.0 to 1.0)	101 (100.0)	10.0 (8.0 to 13.0)
Subtotal	150	150 (100.0)	1.0 (1.0 to 1.0)	150 (100.0)	10.0 (7.0 to 13.0)
**High-Frequency Tone**					
Age (years)					
20–29	69	69 (100.0)	2.0 (1.0 to 2.0)	69 (100.0)	12.0 (10.0 to 17.0)
30–39	53	53 (100.0)	2.0 (1.0 to 2.0)	53 (100.0)	12.0 (9.0 to 14.0)
40–49	28	27 (96.4)	1.0 (1.0 to 2.0)	27 (96.4)	11.0 (8.0 to 14.0)
Sex					
Male	49	48 (98.0)	2.0 (1.0 to 2.0)	48 (98.0)	13.0 (9.0 to 16.0)
Female	101	101 (100.0)	2.0 (1.0 to 2.0)	101 (100.0)	12.0 (10.0 to 14.0)
Subtotal	150	149 (99.3)	2.0 (1.0 to 2.0)	149 (99.3)	12.0 (9.0 to 16.0)
**Total**	**600**	**599 (99.8)**	**1.0 (1.0 to 2.0)**	**599 (99.8)**	**10.0 (8.0 to 13.0)**

*IQR* Interquartile range

**Fig. 1:**
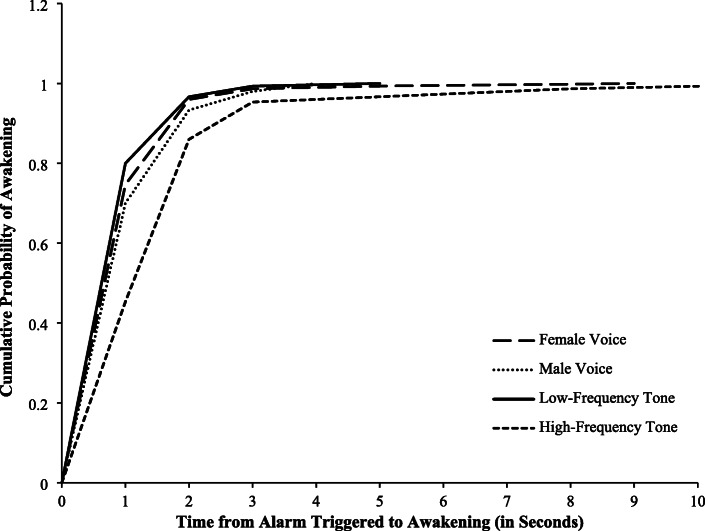
Cumulative Probability of Awakening by Type of Alarm

**Fig. 2:**
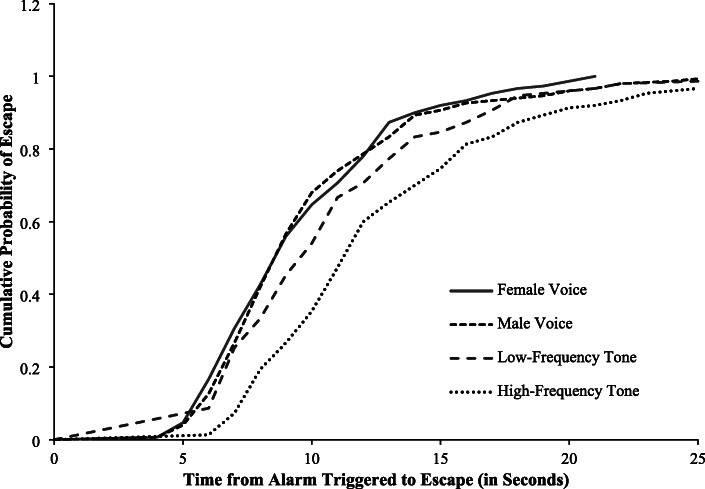
Cumulative Probability of Escape by Type of Alarm. **Note:** Tables and Figures can be placed in the text following the paragraph where reference to the Table or Figure first appears. Final placement will depend on layout by publisher

The cumulative probability of awakening and escaping for the four alarms is shown in Figs. 1 and 2. Overall, the probability functions for time-to-awaken were significantly different (Wilcoxon: *p* < 0.001) for the four alarms. Compared with the high-frequency tone alarm, there were statistically significant differences in the probability functions for time-to-awaken for the female voice alarm (Wilcoxon: *p* < 0.001; HR: 1.40, 95% CI: 1.11–1.77), male voice alarm (Wilcoxon: p < 0.001; HR: 1.33, 95% CI: 1.06–1.68), and low-frequency tone alarm (Wilcoxon: *p* < 0.001; HR: 1.50, 95% CI: 1.19–1.89) (Table 3). However, the differences between the median times-to-awaken were small; the median time-to-awaken was 2.0 s for the high-frequency tone alarm and 1.0 s for the low-frequency tone and each of the voice alarms (Table 2). Similarly, the probability functions for time-to-escape were significantly different for the four alarms overall (Wilcoxon: *p* < 0.001). Time-to-escape probability functions for the female voice alarm (Wilcoxon: p < 0. 001; HR: 1.83, 95% CI: 1.45–2.31), male voice alarm (Wilcoxon: p < 0.001; HR: 1.71, 95% CI: 1.36–2.15), and low-frequency tone alarm (Wilcoxon: p < 0.001; HR: 1.42, 95% CI: 1.13–1.78) were significantly different than the probability function for the high-frequency tone alarm (Table 3). Differences in the median times-to-escape were small; the median time-to-escape for the high-frequency tone alarm was 12.0 s, compared with 10.0 s for the low-frequency tone alarm and 9.0 s each for the female and male voice alarms (Table 2). Pairwise comparisons of the probability functions for time-to-awaken and time-to-escape between the low-frequency tone alarm, female voice alarm, and male voice alarm indicated no statistically significant differences between each of these pairs, except for the comparison of the female voice alarm with the low-frequency tone alarm for time-to-escape (Wilcoxon: *p* = 0.03; HR: 1.30, 95% CI: 1.03–1.63) (Table 3).

**Table 3:** Comparisons of Time-to-Awaken and Time-to-Escape Between Types of Alarms

	Time to Awaken		Time to Escape	
Alarm Signal Comparison	Wilcoxon’s ***P***-Value	HR (95% CI)*	Wilcoxon’s P-Value	HR (95% CI)*
**Overall**				
Equality of Alarm Signals	< 0.001		< 0.001	
**Pairwise Comparison**				
‘Female voice’ vs ‘High-frequency tone’	<.001	1.40 (1.11–1.77)	<.001	1.83 (1.45–2.31)
‘Male voice’ vs ‘High-frequency tone’	<.001	1.33 (1.06–1.68)	<.001	1.71 (1.36–2.15)
‘Low frequency tone’ vs ‘High-frequency tone’	<.001	1.50 (1.19–1.89)	<.001	1.42 (1.13–1.78)
‘Female voice’ vs ‘Male voice’	0.346	1.05 (0.84–1.32)	0.7946	1.07 (0.85–1.35)
‘Female voice’ vs ‘Low frequency tone’	0.340	0.93 (0.74–1.17)	0.0272	1.30 (1.03–1.63)
‘Male voice’ vs ‘Low frequency tone’	0.058	0.89 (0.71–1.11)	0.052	1.21 (0.96–1.52)

*HR* Hazard ratio

*CI* Confidence interval

## Discussion

All alarms performed well in awakening and prompting escape of adult subjects in this study. Only one individual did not awaken or escape to a single alarm (high-frequency tone). Although the statistically significant differences in the probability functions for time-to-awaken and time-to-escape were in favor of the low-frequency tone and voice alarms compared with the high-frequency tone alarm, the differences between alarms were small and not meaningfully different in the context of a real-world residential fire. These findings are different than those for children 5–12 years old in our previous studies, where the performance of the high-frequency tone was clearly inferior to that of the other types of alarms (Tables 4 and 5) (Smith et al. 2019; Smith et al. 2006; Smith et al. 2020a; 2020b). The results of the current study confirm that the female voice, male voice, and low-frequency tone alarms, which are effective for sleeping children, also effectively awaken and prompt escape among sleeping adults. This is important because it is impractical to have separate alarms for children and adults in a household.

**Table 4:** Comparison of Median Time-to-Awaken and Median Time-to-Escape Between Children and Adults

Type of Alarm	Median Time-to-Awaken (seconds)		Median Time-to-Escape (seconds)	
	Children^a^	Adults	Children^**a**^	Adults
Female Voice	4.0	1.0	24.0	9.0
Low-Frequency Tone	4.0	1.0	41.5	10.0
High-Frequency Tone	> 300.0	2.0	> 300.0	12.0

^a^Values for children are from Smith GA, et al. *Academic Pediatrics.* 2019 (2020a)

**Table 5:** Comparison of Time-to-Awaken and Time-to-Escape Between Children and Adults

	Time-to-Awaken		Time-to-Escape	
	Hazard Ratio (95% CI)		Hazard Ratio (95% CI)	
**Pairwise Alarm Signal Comparisons**	**Children**^a^	**Adults**	**Children**^a^	**Adults**
Female voice vs High-Freq tone	2.52 (1.92–3.30)	1.40 (1.11–1.77)	2.76 (2.10–3.62)	1.83 (1.45–2.31)
Low-Freq tone vs High-Freq tone	2.96 (2.27–3.85)	1.50 (1.19–1.89)	2.66 (2.03–3.47)	1.42 (1.13–1.78)

^a^Values for children are from Smith GA, et al. *Academic Pediatrics.* 2019 (2020a)

*CI* Confidence interval

This study supports the development of a smoke alarm for sleeping children by demonstrating the effectiveness of candidate alarms among adults. The development of such an alarm is important because children 5–12 years old have a higher residential fire fatality rate than teenagers and adults up to age 35 years (Ahrens 2014). Although they are potentially capable of self-rescue in a residential fire, they are unlikely to awaken to the high-frequency tone smoke alarm found in many homes (Busby et al. 1994; Smith et al. 2019; Underwriters Laboratories, Smoke alarm STP research group 2003). Our research has contributed to the relatively small literature on this topic (Bruck 1998; Bruck 1999; Bruck and Bliss n.d.; Bruck et al. n.d.; Bruck and Thomas 2012) and employs improvements in study methodology, such as larger sample sizes, monitoring and controlling for sleep stage, using a repeated measures design to mitigate the potential effects of inter-subject variation in AATs (Bruck 2001; Zepelin et al. 1984), and including an escape procedure. Inclusion of an escape procedure is important because a person not only needs to awaken, but also needs to escape in the event of a fire.

Our studies have demonstrated that these alarms do not have to be personalized for effectiveness, such as using a voice message that includes the person’s first name or a familiar voice (like a mother’s voice). This is important because an alarm can be manufactured at a lower cost using a generic recording and can be installed without the effort of personalization by the consumer. The decreased cost and increased ease of installation increases the likelihood that the alarm would be used and installed correctly (Baker 1981).

In a previous study of alarm effectiveness among children 5–12 years old, the low-frequency tone was marginally better at awakening children but had a somewhat longer time-to-escape than the female voice alarm (Table 4) (Smith et al. 2020a). Therefore, hypothesizing that there may be advantages to combining these signals into one alarm and that the message content of the voice alarm may provide valuable instructions regarding life-saving escape behaviors to a child during the period of confusion associated with sleep inertia upon awakening (Smith and Wogalter 2007), we tested a hybrid alarm among 5–12-year-old children (Smith et al. 2020b). The hybrid alarm that combined the low-frequency tone and female voice performed well among children, but has not been tested among adults (Smith et al. 2020b). Testing the effectiveness of the hybrid alarm among adults merits further research. In addition, the voice message used in our studies was designed to awaken individuals and prompt performance of the simulated escape procedure that was used. Potential next steps include convening a panel of fire safety professionals to develop a universal message for use in residential voice alarms.

### Study limitations

This study had some limitations. It was conducted among adults who 1) did not have a medical condition (such as obstructive sleep apnea) and weren’t taking a medication that might affect sleep, arousal, or their ability to perform the study’s escape procedure, 2) did not have a hearing impairment, and 3) did not have an acute illness at the time of the study. In addition, our study did not test smoke alarms among individuals after drinking alcohol. Alcohol use is a known risk factor for fire-related death, and drinking alcohol has been shown to decrease an adult’s ability to awaken to smoke alarms at blood alcohol concentrations of 0.05 and 0.08 (Ball and Bruck 2004). Therefore, our study findings do not apply to all sleeping adults under all conditions. The study also did not include an adaptation night, which is often employed to avoid a “first night effect.” However, such an effect was minimized by the repeated measures study design and by waking study participants from S4S, which is the sleep stage least influenced by potential confounders because of decreased cortical arousability (Bonnet et al. 1978). Participants rehearsed the escape procedure immediately before falling asleep in this study, which may have affected the time-to-escape; however, escape times were brief and demonstrated little variability among these adult subjects.

## Conclusions

All alarms performed well, demonstrating that smoke alarms that were developed for the unique developmental requirements of sleeping children are also effective among sleeping adults. Compared with a high-frequency tone alarm, use of these alarms may reduce residential fire-related injuries and deaths among children while also successfully alerting adult members of the household. Now that optimized smoke alarm signals for children and adults have been identified, future research should test them among older adults, although previous research suggests that they should respond at least as well as adults younger than 65 years old (Bruck et al. 2006).

## Data Availability

The dataset analyzed during the current study is available from the corresponding author on reasonable request for research purposes following completion of publication of findings based on this dataset.

## Abbreviations

AAT: Auditory arousal threshold
CI: Confidence interval
dB: Decibel
EEG: Electroencephalography
HR: Hazard ratio
Hz: Hertz
IQR: Interquartile range
PSG: Polysomnography
S4S: Stage 4 sleep
